# Gait analysis with wearables predicts conversion to Parkinson disease

**DOI:** 10.1002/ana.25548

**Published:** 2019-07-27

**Authors:** Silvia Del Din, Morad Elshehabi, Brook Galna, Markus A. Hobert, Elke Warmerdam, Ulrike Suenkel, Kathrin Brockmann, Florian Metzger, Clint Hansen, Daniela Berg, Lynn Rochester, Walter Maetzler

**Affiliations:** ^1^ Institute of Neuroscience/Newcastle University Institute for Ageing, Clinical Ageing Research Unit, Campus for Ageing and Vitality Newcastle University Newcastle upon Tyne UK; ^2^ Center for Neurology and Hertie Institute for Clinical Brain Research, Department of Neurodegenerative Diseases University Hospital Tübingen, and Center for Neurodegenerative Diseases Tübingen Germany; ^3^ Department of Neurology University Medical Center Schleswig‐Holstein Kiel Germany; ^4^ School of Biomedical Sciences Newcastle University Newcastle upon Tyne UK; ^5^ Geriatric Center and the Department of Psychiatry and Psychotherapy University Hospital Tübingen Tübingen Germany; ^6^ Newcastle upon Tyne University Hospitals National Health Service Foundation Trust Newcastle upon Tyne UK

## Abstract

**Objective:**

Quantification of gait with wearable technology is promising; recent cross‐sectional studies showed that gait characteristics are potential prodromal markers for Parkinson disease (PD). The aim of this longitudinal prospective observational study was to establish gait impairments and trajectories in the prodromal phase of PD, identifying which gait characteristics are potentially early diagnostic markers of PD.

**Methods:**

The 696 healthy controls (mean age = 63 ± 7 years) recruited in the Tubingen Evaluation of Risk Factors for Early Detection of Neurodegeneration study were included. Assessments were performed longitudinally 4 times at 2‐year intervals, and people who converted to PD were identified. Participants were asked to walk at different speeds under single and dual tasking, with a wearable device placed on the lower back; 14 validated clinically relevant gait characteristics were quantified. Cox regression was used to examine whether gait at first visit could predict time to PD conversion after controlling for age and sex. Random effects linear mixed models (RELMs) were used to establish longitudinal trajectories of gait and model the latency between impaired gait and PD diagnosis.

**Results:**

Sixteen participants were diagnosed with PD on average 4.5 years after first visit (converters; PDC). Higher step time variability and asymmetry of all gait characteristics were associated with a shorter time to PD diagnosis. RELMs indicated that gait (lower pace) deviates from that of non‐PDC approximately 4 years prior to diagnosis.

**Interpretation:**

Together with other prodromal markers, quantitative gait characteristics can play an important role in identifying prodromal PD and progression within this phase. ANN NEUROL 2019;86:357–367

Parkinson disease (PD) is a progressive neurodegenerative disorder including a prodromal period during which the disease has started but the definitive motor and nonmotor symptoms to permit a diagnosis have not yet appeared.^1^, ^2^, ^3^ Identifying people at this stage is of significant interest, as this is the time in which neuroprotective therapies should be delivered, when they become available.

Previous research has tried to characterize the prodromal interval of PD in terms of features and duration. This prodromal PD phase has been estimated by previous retro‐ and prospective studies to be between 3 and 15 years, with this period varying depending on type of marker and patient‐related factors such as age.^4^, ^5^ Utilization of clinical scales^6^ (ie, the Unified Parkinson Disease Rating Scale [UPDRS]) and neuropathological studies (based on cell loss in the substantia nigra) suggest a prodromal period of approximately 5 years.^7^

Idiopathic rapid eye movement sleep behavior disorder (RBD) is a particularly interesting prodromal marker. More than 50% convert to PD or similar diseases (such as dementia with Lewy bodies) with an average duration of the overall prodromal phase of 13 years^8^ and a motor phase of 4.5 years.^9^, ^10^, ^11^ A study using simple methods for description of motor abnormalities (UPDRS and Timed Up and Go test) suggests that first abnormalities in gait precede diagnosis by up to 4.4 to 6.3 years.^10^ A quantitative cross‐sectional evaluation of normal‐pace walking in RDB participants found subtle changes of velocity, cadence, and variability of gait compared to controls.^12^ Comparable results were found in a quantitative cross‐sectional evaluation of gait—here, however, only during challenging (dual‐task) conditions—in LRRK2 mutation carriers who showed increased gait variability and decreased amplitude of the dominant peak of the accelerometer signal.^13^ Another cross‐sectional study^14^ showed increased arm‐swing variability in LRRK2 mutation carriers that was basically visible in non‐PD and PD state under challenging conditions (ie, dual‐task conditions). These latter studies therefore cannot differentiate between initial abnormalities in the central gait network of RBD and LRRK2 cohorts (which can be entirely independent of dynamic processes happening in a prodromal PD phase) and “real” prodromal PD markers, thus highlighting the need for prospective longitudinal and population‐based studies.

The use of wearable sensors to measure gait is showing significant promise.^15^, ^16^, ^17^, ^18^, ^19^, ^20^

The aim of this longitudinal prospective observation study over 6 years in a cohort of healthy controls (HCs) was therefore to investigate whether a comprehensive set of gait characteristics measured with wearable technology, that is, a wearable device, can define prodromal PD by (1) evaluating if gait characteristics can predict risk of conversion, (2) estimating the year‐on‐year change in gait characteristics, and (3) identifying the time at which changes in gait would start being evident prior to diagnosis. We hypothesized that (1) selected gait characteristics predict future PD conversion, (2) gait declines continuously during the prodromal phase, and (3) first gait changes in future PD converters (PDCs) appear 4 to 5 years prior to diagnosis.

## Subjects and Methods

### *Participants*

Data were collected longitudinally at 2‐year intervals between 2009 and 2016 (T1–T4). Six hundred and ninety‐six HCs recruited in the Tubingen Evaluation of Risk Factors for Early Detection of Neurodegeneration (TREND) study were assessed.^21^, ^22^ Participants were included in the study if they were aged 50 to 80 years and were excluded if they had any orthopedic or cardiothoracic conditions that might have markedly affected their walking or safety during the testing sessions. To maximize generalizability of the results, we included the whole TREND cohort.

### *Ethics, Consent, and Permissions*

The ongoing TREND study is being performed by the Departments of Neurology and Psychiatry of the University Hospital Tübingen. It is being conducted according to the declaration of Helsinki and has ethical approval from the ethics committee of the Medical Faculty, University of Tübingen (90/2009BO2). All participants signed an informed consent form prior to testing.

### *Demographic and Clinical Measures*

Age, sex, body mass index (BMI), years of education (defined by the International Standard Classification of Education^23^) Beck Depression Inventory I score,^24^ and self‐reported depression^25^ were recorded for each participant. The Movement Disorders Unified Parkinson's Disease Rating Scale part III (MDS‐UPDRS‐III), Mini‐Mental State Examination, and Consortium to Establish a Registry for Alzheimer's Disease (CERAD)^26^ were among various tests in the assessment battery.^22^ Tremor was also defined retrospectively based on MDS‐UPDRS‐III following Bain,^27^ considering what the movement specialists evaluated as more than physiological and mild tremors during a TREND visit and persisting in the following TREND visit(s). Inclusion criteria of the TREND study were being a healthy adult older than 50 years and being free of significant hearing or visual impairments. Exclusion criteria were a diagnosis of a neurodegenerative disease, stroke, or inflammatory central nervous disease or the administration of dopaminergic or antipsychotic drugs.

The prodromal PD probability score was calculated for all study visits according to Berg et al.^1^ The following parameters were used: age for the pretest probability and for the posttest probability, risk markers (sex, smoking status, family history for PD, and substantia nigra hyperechogenicity status),^28^ and prodromal markers (subthreshold parkinsonism defined as a MDS‐UPDRS‐III total score above 6 points excluding postural and action tremor), RBD assessed by the RBD screening questionnaire,^29^ hyposmia assessed by the Sniffin’ Sticks test battery^30^ using normative values,^31^ depression (self‐reported history of major depression or acute major depression^25^), symptomatic orthostatic hypotension, urinary dysfunction, erectile dysfunction, and constipation assessed by the Unified Multiple System Atrophy Rating Scale.^32^

### *Laboratory Data Collection: Equipment and Gait Protocol*

**Figure 1:**
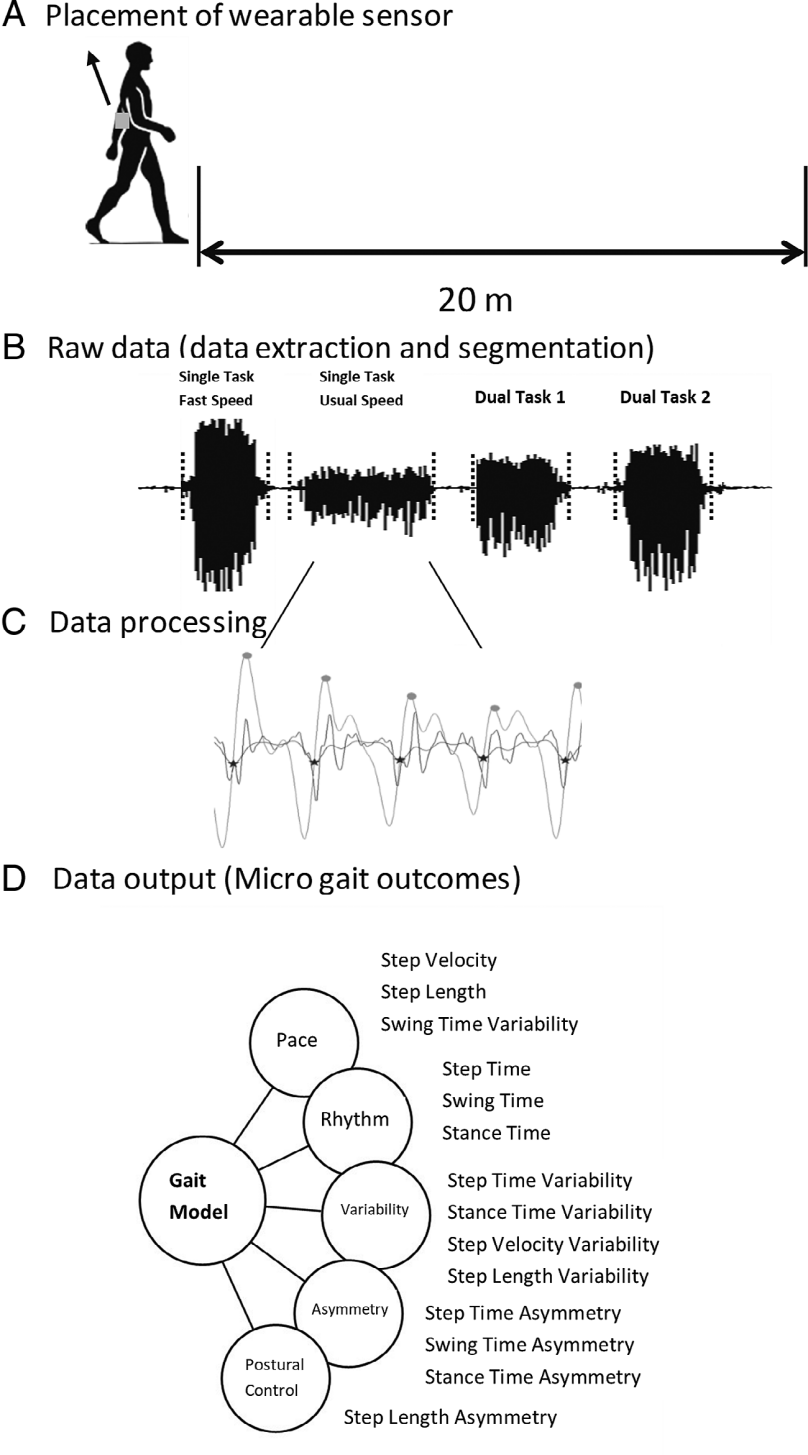
Clinic assessment of gait characteristics: (A) Example of body worn monitor placement and setup for walking data collection. (B) Raw vertical acceleration processing: data extraction and segmentation (signal segments in *dotted black lines*) from walking conditions (in order: single task at fast speed, single task at usual speed, dual task 1, and dual task 2). (C) Example of gait characteristic evaluation from a walking bout. (D) Data output: conceptual model of gait representing 5 domains and 14 gait characteristics.

Each participant wore a device, either DynaPort (McRoberts, the Hague, the Netherlands; 100 Hz; T1, T2) or Opal (APDM, Portland, OR; 128Hz; T3, T4), located on the area of the fourth and fifth lumbar vertebrae (L4, L5; Fig 1A). Participants were asked to perform intermittent straight line walking trials over a 20m walkway under 4 different conditions: (1) single task at their usual (preferred) speed, (2) single task at fast speed, (3) dual task at fast speed with checking boxes (participants marked as many boxes as possible on a sheet of paper on a clipboard as fast as possible with a cross using a pen, defined as dual task 1), and (4) dual task at fast speed with serial subtractions (counting backward in sevens, defined as dual task 2; Fig 1B).^21^

Fourteen gait characteristics comprising 5 domains (pace, variability, rhythm, asymmetry, and postural control; Fig 1C, D), representative of a conceptual model of gait,^33^ were evaluated from the accelerometer signals with a validated gait analysis algorithm from our group, which are extensively described.^34^, ^35^ Briefly, triaxial acceleration signals were downloaded to a computer, segmented into individual walking trials using time stamps, and analyzed by a bespoke MATLAB (R2015a) program (see Fig 1B). All 128Hz data (APDM) were down‐sampled to 100Hz (frequency of the Dynaport). Accelerometer signals were transformed to a horizontal–vertical coordinate system^36^ and filtered with a fourth‐order low pass Butterworth filter at 20Hz.^37^, ^38^, ^39^ The initial contact (IC; heel strike) and final contact (FC; toe off) events within the gait cycle were identified from the Gaussian continuous wavelet transform of the vertical acceleration. IC and FC detection allowed the estimation of step, stance, and swing time.^34^ The IC events were also used to estimate step length using the inverted pendulum model.^39^ To estimate a value for step velocity, we utilized the simple ratio between step distance (length) and step time.^34^ To calculate variability characteristics (eg, step time variability), the standard deviation (SD) from all steps (left and right combined) was calculated. Asymmetry characteristics (eg, step time asymmetry) were determined as the average absolute difference between left and right steps (alternating) for each walking pass.^34^

### *Statistical Analysis*

Statistical analysis was carried out using SPSS v24. Normality of data and homoscedasticity were tested with Shapiro–Wilk test and Levene's test of equality of variances, respectively. Descriptive statistics were reported as means and SDs.

The analysis had 3 key parts, according to our initially mentioned aims and hypotheses.

#### *Defining the Predictive Value of First Visit Gait Characteristics to Identify Risk of PD Conversion*

For this purpose, Cox regression was performed, controlling for age at first visit and sex (male, female) entered as an initial block, before entering the gait characteristics in block 2. BMI and years of education at first visit were considered as covariates; however, they did not contribute significantly and so were not included in the final model. After considering each gait characteristic individually, we computed both backward and forward conditional models to examine whether a combination of gait characteristics could better predict conversion to PD. Age at first visit and sex were again entered in the first block, and then all gait variables were entered in the second block. Given the exploratory nature of this analysis, we used a threshold of *p* < 0.05 to guide statistical interpretation and did not make adjustments for multiple comparisons.^12^, ^40^, ^41^ However, we provide the *p* value for each comparison so that the reader may assess the statistical strength of our findings.

#### *Estimate the Yearly Change for Each Gait Characteristic*

For this purpose, random effects linear mixed models were used. The intercept and yearly slope were modelled as random effects, using a diagonal covariance structure. Sex was modelled as a fixed effect. For non‐PDC, age was centered about the mean PDC group age at first visit. For PDC, age was centered about their individual date of diagnosis. *T* tests (Welch variant to accommodate unequal variance) were used to test for differences in gait at the time of PD diagnosis and yearly change in gait. Data collected after PD diagnosis was excluded for longitudinal analysis to avoid the potential confounding effects of medication and clinical management on gait.

#### *Estimate How Many Years prior to Diagnosis Gait of PDC Differed from Non‐PDC*

For this purpose, we used a recently published method^10^ that divided the difference in gait between non‐PDC and PDC at the time of diagnosis by the yearly change of the PDC. To reduce the number of models, we focused on gait characteristics which changed over time in the PDC group in conjunction with a group difference at the time of PD diagnosis. To accommodate for unequal proportion of females between the groups, estimated means were calculated assuming 50% of males and females in both groups.

## Results

Clinical, demographic, and cognitive descriptors of the whole TREND study cohort are shown in Table 1. Retention rates with respect to first visit (T1) were 90.9% at T2, 83% at T3, and 76.5% at T4. A higher probability of prodromal PD score in the control cohort at first visit was associated with dropping out of the study sooner; however, this association became nonsignificant after controlling for age at first visit and sex (*p* = 0.129). Among the 696 participants, 16 were diagnosed with PD on average 4.5 years after the first visit (future PDCs) and 680 remained non‐PDC. For descriptive purposes only, we report details of the PDC and non‐PDC groups in the Supplementary Table.

**Table 1:** Clinical and Demographic Characteristics for the Whole TREND Cohort, N = 696

Characteristic	Value
Female, n (%)	378 (54)
Age, yr, mean (SD)	63 (7)
BMI, kg/m^2^, mean (SD)	26 (4)
Education, yr, mean (SD)	15 (3)
MMSE, 0–30, mean (SD)	29 (1)
CERAD, 0–100, mean (SD)	85 (7)
Probability score mean (SD)	7 (14)
Tremor, n (%)	35 (5)
BDI‐I score mean (SD)	8 (7)
Self‐reported depression, n (%)	265 (38)

Probability score indicates individual probability of prodromal Parkinson disease (PD) score, with inclusion of the following features: substantia nigra hyperechogenicity, probable rapid eye movement sleep behavior disorder, subthreshold parkinsonism, hyposmia, erectile dysfunction, constipation, and PD family history.^28^

BDI‐I = Beck Depression Inventory I; BMI = body mass index; CERAD = Consortium to Establish a Registry for Alzheimer's Disease neuropsychological battery^26^; MMSE = Mini‐Mental State Examination^50^; SD = standard deviation; TREND = Tubingen Evaluation of Risk Factors for Early Detection of Neurodegeneration.

### *Predicting PD Conversion with Gait Characteristics*

Descriptive data for each gait characteristic under normal walking conditions for the whole cohort are shown in Table 2.

**Table 2:** Prediction of PD Conversion by Gait Characteristics

Predictors	Gait Characteristics at T1	Base Model	*p*	Forward Stepwise Model		Backward Stepwise Model	
		β (CI 95%)		β (CI 95%)	*p*	β (CI 95%)	*p*
Sex, F		−1.7 (−3.3 to −0.2)	0.025^a^	−2.0 (−3.5 to −0.4)	0.013^a^	−2.4 (−4.2 to −0.7)	0.007^a^
Age		0.088 (−0.003 to 0.179)	0.058	0.083 (−0.013 to 0.180)	0.091	0.100 (−0.004 to 0.204)	0.058
Pace							
Step velocity, m/s	1.173 (0.162)	−1.1 (−4.7 to 2.5)	0.561				
Step length, m	0.588 (0.066)	−5.5 (−15.2 to 4.3)	0.274				
Swing time Var, s	0.021 (0.014)	27.6 (2.2 to 52.9)	0.033^a^				
Variability							
Step velocity Var, m/s	0.088 (0.040)	4.2 (−8.4 to 16.9)	0.511			−16.6 (−36.6 to 3.4)	0.103
Step length Var, m	0.035 (0.016)	21.4 (−10 to 52.9)	0.181				
Step time Var, s	0.026 (0.017)	24.4 (2.7 to 46)	0.028^a^			50.9 (12.5 to 89.3)	0.009^a^
Stance time Var, s	0.024 (0.014)	24.5 (−2.8 to 51.9)	0.079				
Rhythm							
Step time, s	0.507 (0.043)	0.3 (−8.5 to 9.2)	0.938			−17.1 (−34.9 to 0.7)	0.060
Swing time, s	0.353 (0.037)	1.8 (−13.9 to 17.4)	0.826				
Stance time, s	0.660 (0.057)	−3.7 (−14.3 to 7)	0.499				
Asymmetry							
Step time Asy, s	0.032 (0.032)	16.5 (5.6 to 27.5)	0.003^a^				
Swing time Asy, s	0.025 (0.025)	20.2 (7.2 to 33.2)	0.002^a^	17.3 (2.9 to 31.8)	0.019^a^		
Stance time Asy, s	0.025 (0.025)	19.7 (6.4 to 33.0)	0.004^a^				
Postural control							
Step length Asy, m	0.025 (0.021)	29.8 (14.8 to 44.9)	<0.001^a^	26.4 (10.9 to 41.9)	0.001^a^	29.6 (13.3 to 45.8)	<0.001^a^

Cox regression predicting time (in months) to PD conversion for individual gait characteristics (base model) and for the combination of gait characteristics (forward and backward stepwise regression). Age and sex were entered into the first block prior to entering gait characteristics in the second block. Gait characteristics are presented for the whole Tubingen Evaluation of Risk Factors for Early Detection of Neurodegeneration cohort (n = 696) for a single task at the usual‐speed walking condition.

a *p* < 0.05.

Asy = asymmetry; CI = confidence interval; F = female; PD = Parkinson disease; Var = variability; β = unstandardized beta values where a larger beta value refers to an increase in hazard and so a shorter time to diagnosis.

**Figure 2:**
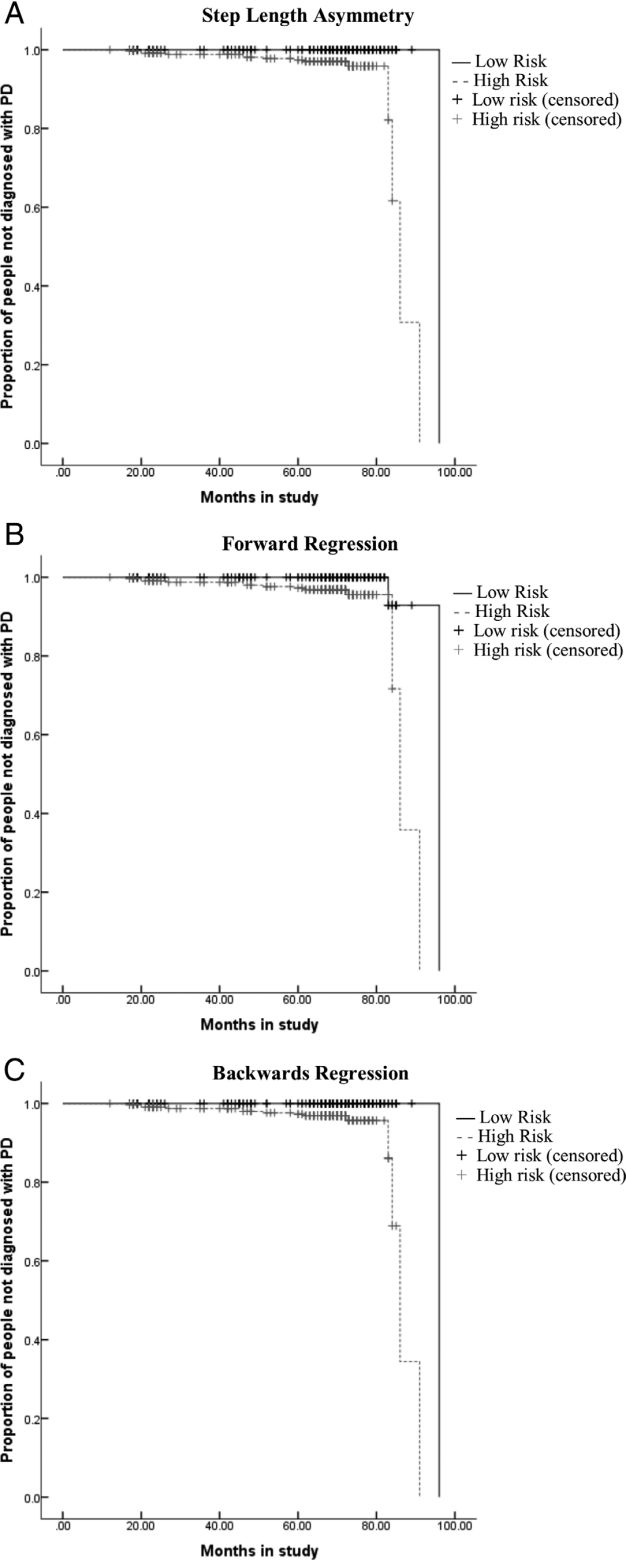
Kaplan–Meier curves illustrating shorter time to Parkinson disease (PD) diagnosis using different combinations of first visit gait variables derived from univariate analysis and forward and backward stepwise Cox regression. (A) Greater step length asymmetry at first visit (adjusted for age and sex). (B) Combination of greater step length and swing time asymmetry (adjusted for age and sex). (C) Combination of greater step length asymmetry, higher variability of step velocity and step time, and quicker step time (adjusted for age and sex). Low and high risk were determined as higher than (high risk) and lower than (low risk) the hazard models created from models described in Table 2.

Cox regression revealed that, during single task at usual‐speed walking, greater gait variability (step and swing time) and asymmetry (step, swing, and stance time and step length) significantly predicted PD conversion. Forward stepwise Cox regression showed the combination of older age, male sex, and more asymmetric swing time and step length best predicted conversion to PD (see Table 2). Backward stepwise Cox regression resulted in a larger combination of predictors (older age, male sex, quicker step time, greater variability of step time and step length, and more asymmetric step length; Fig 2). We repeated the analysis for the remaining walking tasks: for single task at fast speed and dual task 2 walking conditions, only a higher step length asymmetry significantly predicted a shorter time to PD conversion (*p* ≤ 0.018); for dual task 1 walking condition, none of the variables significantly predicted PD conversion. We also checked for correlation between the gait variability and asymmetry and substantia nigra hyperechogenicity, which showed *R* ^*2*^ values below 0.12 (data not shown). In light of these results, we focused on single task at usual‐speed walking for subsequent analyses. Results of the other walking conditions are not reported but are available upon request.

### *Change in Gait Characteristics over Time*

In PDC, over time, step velocity and step length reduced, but step time, stance time, and stance time asymmetry increased (Table 3).

**Table 3:** Yearly Change of Gait Characteristics of the PDCs

Single Task at Usual Speed	PDC Intercept (SD)^a^	PDC Yearly Change (SD)
Pace		
Step velocity, m/s	1.007 (0.359)	−0.0295 (0.0353)^b^
Step length, m	0.514 (0.158)	−0.0134 (0.0138)^b^
Swing time Var, s	0.040 (0.032)	0.0021 (0.0004)
Variability		
Step velocity Var, m/s	0.125 (0.057)	0.0021 (0.0101)
Step length Var, m	0.047 (0.036)	0.0023 (0.0045)
Step time Var, s	0.043 (0.037)	0.0008 (0.0056)
Stance time Var, s	0.042 (0.031)	0.0021 (0.0043)
Rhythm		
Step time, s	0.514 (0.083)	0.0028 (0.0034)^b^
Swing time, s	0.371 (0.062)	0.0015 (0.0067)
Stance time, s	0.663 (0.119)	0.0044 (0.0067)^b^
Asymmetry		
Step time Asy, s	0.040 (0.065)	−0.0032 (0.0081)
Swing time Asy, s	0.041 (0.063)	−0.0001 (0.0046)
Stance time Asy, s	0.044 (0.062)	0.0006 (0.0006)
Postural control		
Step length Asy, m	0.038 (0.079)	−0.0008 (0.0042)

Intercept and yearly change data for gait characteristics of the PDCs for a single task in the usual‐speed walking condition. Values are presented as mean (standard deviation) and estimated for an equal proportion of males and females in each group.

a At diagnosis.

b Within‐group yearly change of gait *p* < 0.05.

Asy = asymmetry; PDC = Parkinson disease converter; SD = standard deviation; Var = variability.

### *Determining Onset of Change in Gait Characteristics prior to PD Conversion*

**Figure 3:**
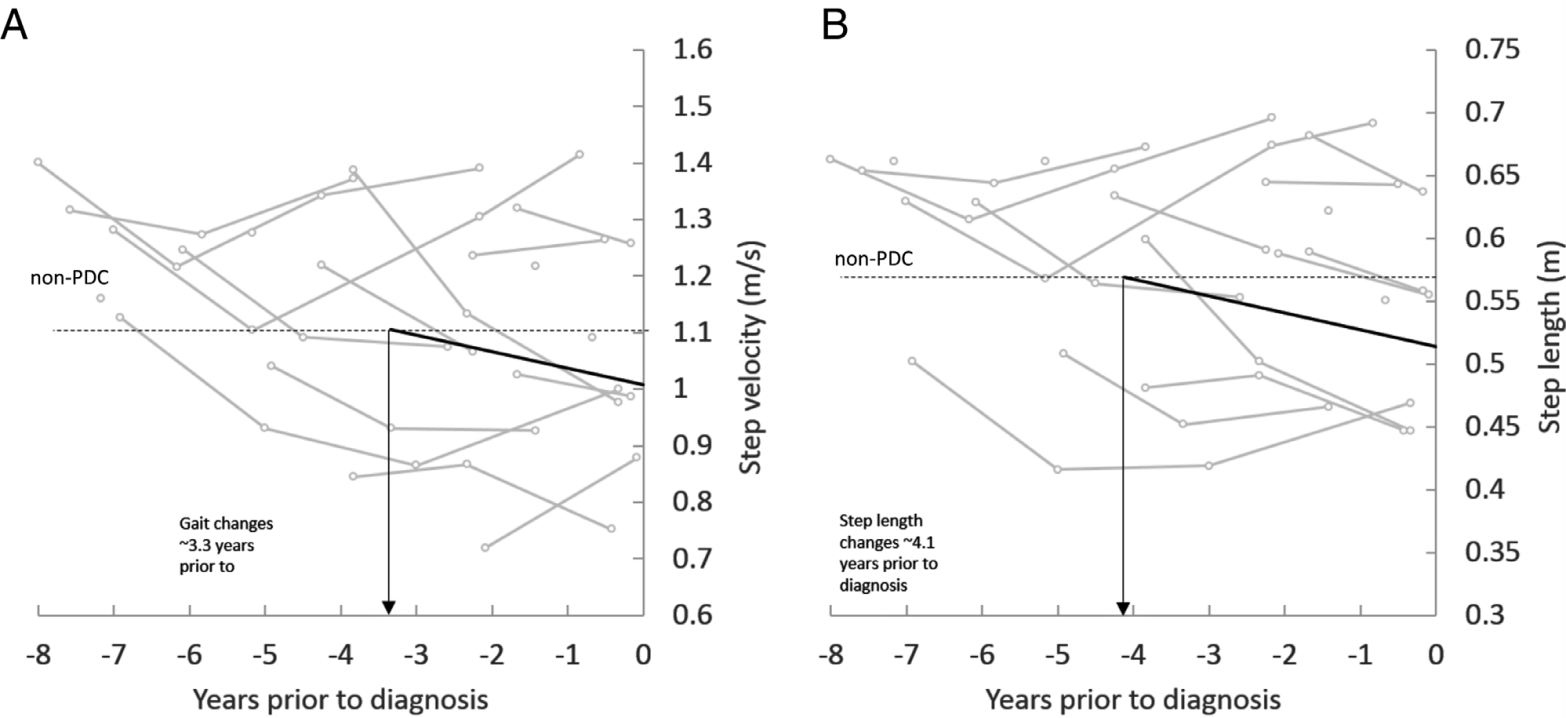
The graph illustrates step velocity and step length at usual speed prior to diagnosis of Parkinson disease (Parkinson disease converters [PDC]; gray lines indicate individuals, solid black line indicates PDC group mean) compared to the mean walking speed of non‐PDC at first visit (indicated by the *dotted line*). The 2 lines intersect approximately 3.3 years prior to PD diagnosis for gait velocity and 4.1 years for step length, indicating the latency between early changes of gait and diagnosis.

Step velocity and step length were the only variables that demonstrated significant change over time in the group of people who subsequently converted to PD using the non‐PDC as a reference, in conjunction with a group difference at the time of PD diagnosis (all pace and variability characteristics). Therefore, step velocity and step length were chosen to estimate how early the gait of PDC deviated from “normal” (ie, non‐PDC). Step velocity deviated from non‐PDC ∼3.3 years (95% confidence interval [CI] = 2.0–9.3) and step length deviated from non‐PDC ∼4.1 years (95% CI = 2.7–9.15) prior to diagnosis of PD (Fig 3). We recalculated these estimates controlling the decline of step velocity and length over time in the non‐PDC, resulting in 1.7 years latency for gait velocity and 3.1 years for step length.

## Discussion

This is the first study to our knowledge to comprehensively examine gait in a cohort of older adults at risk of converting to PD and to carry out longitudinal follow‐up to conversion. We were able to show that gait discriminates future PD converters from HCs up to approximately 4 years prior to clinical diagnosis. Moreover, we show that gait characteristics have the potential to predict conversion to PD and can relevantly add to a panel of prodromal markers for the definition of persons at substantially increased risk for PD. To ensure robustness of the findings also with respect to cognition, we compared results of an extensive neuropsychological test (CERAD) between 2 investigated cohorts, with results that were not significantly different, and we controlled for years of education in our analysis. Our study presents data obtained from wearable technology (1 sensor at the lower back) and validated gait analysis algorithms, which renders this approach a feasible and useful paradigm even for screening in large cohorts and general practice.

### *Can Gait Predict PD Conversion?*

A novel aspect of this study is the longitudinal assessment of gait impairments, associated with PD prior to the onset of clinically significant motor deficits and diagnosis, with a wearable device during a series of walking tasks. This method presented a unique opportunity to observe whether gait could predict risk of PD conversion.

We hypothesized that selected gait characteristics would predict PD conversion.

Indeed, Cox regression indicated that greater gait variability (step and swing time) and asymmetry (step, swing, and stance time and step length) significantly predicted a shorter time to PD conversion during the single task at usual‐speed walking conditions. Only step length asymmetry (fast speed and dual task 1 tasks) significantly predicted a shorter time to PD conversion in the more challenging walking conditions, including dual tasking. This observation is interesting in the light of previous studies focusing on gait alterations in cohorts with potentially increased risk for PD.^13^

To the best of our knowledge, only cross‐sectional studies are currently published reporting about the potential of quantitative gait characteristics; some found differences between persons with increased risk of developing PD and controls under self‐selected gait speed,^12^, ^14^ and some found differences between populations at risk and controls only under more challenging conditions (eg, dual task).^13^, ^14^ This difference with respect to our findings may be due to a different population being investigated (eg, nonmanifesting LRRK2‐G2019S mutation carrier vs PDC) and a distinct dual task protocol (we investigated dual task at fast speed rather than preferred speed like previous studies) and also gait focus (upper limbs vs lower limbs).

Whatever the reasons are for the previously mentioned observations, we provide here the first strong evidence that self‐selected pace may be a promising gait paradigm for the investigation of the prodromal PD phase and of the risk of PD conversion.

Interestingly we found that step length asymmetry was the best single predictor in the regression model during usual (preferred)‐speed walking. This is in line with the known impaired ability to regulate alternate (left/right) steps in PD, and gait asymmetry has been shown to be a sensitive measure of gait (in)stability.^13^, ^14^, ^15^ At the same time, the asymmetric nature of PD is evidently important in detecting early risk. The higher asymmetry (poorer performance) of PDC and the fact that step time asymmetry strongly predicts risk of PD would corroborate the theory that subtle changes in gait asymmetry could reflect prodromal motor alterations, reduced ability of compensatory strategies, and a possible early manifestation of alterations to the central gait network in PDC.^13^

Our results support the asymmetric nature, even before diagnosis, of PD, in agreement with previous work showing that increased gait asymmetry is typical in PD gait.^42^, ^43^, ^44^

In agreement with our findings, previous work involving people at increased risk for PD (RBD patients) showed an association between PD risk and gait variability.^12^ This is an interesting finding when thinking about the typically more variable gait observed in PD patients and the importance given to gait variability measures in PD and disease progression,^45^, ^46^, ^47^ also when quantified with wearable technology.^48^ Thus, these results add to the debate of whether higher gait variability may be “good” or “bad” depending on observed populations (eg, PD, HC, fallers, etc); in this case, our study would corroborate the “badness” of higher variability, potentially representing the start (or the worsening) of an impaired control of gait.

In contrast with previous work, our study found that step velocity was not a significant predictor of PD conversion; this could be due to the different population observed (RBD vs PDC), walking protocol (10m vs 20m walk), and measurement tools used (instrumented mat vs wearable device).^12^

Although we focused on gait only and did not look at arm swing during gait, our results partly support the finding that higher gait asymmetry and variability (especially during single task at usual‐speed walking condition, less heightened during dual task conditions) appear to be prodromal markers for PD.^14^ Together these findings show specific differences in gait characteristics that inform a nuanced understanding of gait changes during the prodromal PD stage.

### *Does Gait Change over Time in PD Converters?*

Our second hypothesis was that gait changes in PDC would decline continuously during the prodromal phase.

We found that over time, walking speed and step length were reduced in both groups, but variability increased. This finding is in agreement with previous studies that looked at gait progression in PD and found that pace (step length) decreased over both shorter (ie, 18 months) and longer (ie, 36 months)^49^ periods of time.

### *When Does Gait Start to Change before Conversion to PD?*

Finally, we hypothesized that, based on previous literature,^10^ first gait changes in PDC appear 4 to 5 years prior to diagnosis. Our study confirms and complements the demonstrated evidence that, in at risk groups, there are subtle changes in gait prior to the onset of significant motor symptoms and these may start about 4 years prior to PD diagnosis.^11^, ^12^ Although we used a different protocol and tools compared to previous studies (UPDRS and Timed Up and Go test),^10^ we found comparable results.

### *Clinical Implications: Cross‐sectional versus Progression Gait Characteristics in Prodromal PD*

Our results show promising cross‐sectional (higher variability and asymmetry) and progression (step velocity and step length) gait characteristics for the detection of the prodromal PD phase. The different nature of these characteristics is in our view not surprising, as they represent different concepts. Cross‐sectional gait characteristics serve as trait markers, to highlight people “at risk” of PD conversion, whereas progression markers (mainly) depend on the intra‐individual dynamic of the characteristic over time. We speculate that combining these characteristics has even more potential than single parameters to identify people at risk for clinically overt PD.

### *Limitations*

Although this is, to our best knowledge, the first prospective longitudinal study on gait characteristics in prodromal PD, we acknowledge that use of different wearable devices may have impacted the variability of the data, and the limited number of PDCs may affect generalizability of the results. Only 1 model of gait characteristics was included in this work; in the future, other reported models and features should be considered to identify the best measure (or combination of measures) for risk prediction and clinical decision‐making. In addition, subtle changes in the prodromal stages of gait necessarily result in wide CIs when estimating how long prior to diagnosis changes in gait occur.

Even though current assumptions about clinically overt and prodromal stages of PD suggest that any manifestations that are nonmotor are “prodromal,” it is possible that the gait abnormalities ascertained here correspond to overlooked motor aspects falling beyond the resolution of clinicians; they may, in a future in which quantitative sensor‐based gait assessments may be widespread, be reclassified as early motor PD.

Even if our findings are consistent with previous work, the combination of greater numbers of converters and more complex nonlinear statistical models (not feasible here given the relatively moderate number of converters) may yield more robust estimates.

We found that gait variability and asymmetry characteristics seem to be the best predictors for the definition of persons in this vulnerable phase before conversion to clinical PD. Interestingly, we obtained the best results from self‐selected walking speed conditions without dual tasking. Step velocity and step length seem to be the best and most stable gait characteristics defining progression during the prodromal PD phase.

Our results suggest that quantitative sensor‐based gait assessment may be considered part of an assessment battery for definition of prodromal PD or with subclinical motor manifestations.

## Author Contributions

W.M., D.B., L.R., and S.D.D. contributed to study concept and design. S.D.D., M.E., B.G., M.H., U.S., K.B., F.M., C.H., and E.W. contributed to data acquisition and analysis. S.D.D., B.G., and M.E. contributed to drafting the manuscript and figures.

## Potential Conflicts of Interest

Nothing to report.

## Supporting information


**Supplementary Table** Clinical and demographic characteristics at first visit of those who converted to Parkinson's disease after a mean period of 4.5 years (PDC) and those who did not (non‐PDC).
