# Supplementary material for: Gait analysis with wearables predicts conversion to Parkinson disease
Source: Ann Neurol. 2019 Jul 27;86(3):357–67. doi: 10.1002/ana.25548 (PMC6899833; doi:10.1002/ana.25548)
Supplement: Supplementary file 1 — Supplementary Table Clinical and demographic characteristics at first visit of those who converted to Parkinson's disease after a mean period of 4.5 years (PDC) and those who did not (non‐PDC). [file ANA-86-357-s001.docx]

**Supplementary Table**

Clinical and demographic characteristics at first visit of those who converted to Parkinson’s disease after a mean period of 4.5 years (PDC) and those who did not (non-PDC).

| **Characteristic** | **PDC**  **(n = 16)** | **Non-PDC**  **(n = 680)** | ***p*** |
| --- | --- | --- | --- |
|  | **Mean (SD)** | **Mean (SD)** |  |
| Female (n, %) | 3 (19%) | 375 (55%) | **0.002** |
| Age [years] | 69 (5) | 63 (7) | **<0.010** |
| BMI [kg/m^2^] | 25 (2) | 26 (4) | 0.854 |
| Years of Education | 15 (3) | 15 (3) | 0.976 |
| MMSE (0-30) | 28 (1) | 29 (1) | **0.003** |
| CERAD (0-100) | 86 (5) | 85 (7) | 0.620 |
| Months to Diagnosis | 55 (28) | - | - |
| Probability Score [%] | 36 (33) | 6 (12) | **<0.001** |
| Tremor (n, %) | 2 (13%) | 33 (5%) | 0.876 |
| BDI-I Score | 7 (6) | 8 (7) | 0.270 |
| Self-reported Depression (n, %) | 5 (31%) | 260 (38%) | 0.414 |

Values are presented as mean (standard deviation) if not otherwise indicated. Significant p-values (p < 0.05) from Cox-regression analysis are presented in bold. BDI-I Score, Beck Depression Inventory I Score; BMI, Body Mass Index; CERAD, The Consortium to Establish a Registry for Alzheimer's Disease neuropsychological battery^26^; MMSE, Mini–Mental State Examination^50^; Probability Score, individual probability of prodromal Parkinson`s disease (PD) score, with inclusion of the following features: substantia nigra hyperechogenicity, probable REM sleep behaviour disorder, subthreshold parkinsonism, hyposmia, erectile dysfunction, constipation & PD family history^28^.
